# Computer vision syndrome among high school students using digital versus printed textbooks: prevalence and associated factors

**DOI:** 10.3389/fpubh.2026.1957177

**Published:** 2026-10-14

**Authors:** Mar Seguí-Crespo, Mar Sánchez-Brau, José Miguel Martínez, Ana Tauste, Eva Artime-Ríos, Elena Ronda-Pérez

**Affiliations:** 1 Department of Optics, Pharmacology and Anatomy, University of Alicante, San Vicente del Raspeig, Spain; 2 Public Health Research Group, University of Alicante, San Vicente del Raspeig, Spain; 3 School of Public Health, University of Alberta, Edmonton, AB, Canada; 4 Research Group in Preventive Optometry and Visual Treatments - OPTV, Department of Optics and Optometry and Vision Sciences, Universitat de València, Burjassot, Valencia, Spain; 5 Occupational Health Service, Central-Southwestern Healthcare Area II, Public Health Service of the Principality of Asturias (SESPA), Oviedo, Spain; 6 Biomedical Research Networking Center for Epidemiology and Public Health (CIBERESP), Madrid, Spain

**Keywords:** adolescent, computer vision syndrome, digital devices, questionnaire, textbook

## Abstract

**Background:**

The increasing implementation of digital textbooks in schools raises concerns regarding their potential impact on the ocular and visual health of adolescents. However, the effect of this educational shift on computer vision syndrome (CVS) has not yet been assessed using validated measurement tools.

**Objective:**

This study analyses the prevalence of CVS in adolescent students, comparing the use of printed versus digital textbooks, and considering sociodemographic, visual and digital devices exposure factors.

**Methods:**

A longitudinal observational study with repeated assessments of the same participants and estimation of CVS at four time points was conducted during the second academic semester in two high schools in Alicante, Spain (*n* = 231 students, 15–17 years old). One high school used printed textbooks, whereas the other implemented digital textbooks. An initial visual examination was performed, assessing visual acuity, objective and subjective refraction, eye movements, pupillary reflexes, and the cover test. At each visit, participants completed a questionnaire on digital device exposure and together with the validated CVS-Q© teen questionnaire. Generalized Estimating Equation logistic regression models were used to estimate crude and adjusted odds ratios (aOR) and 95% confidence intervals (95% CI).

**Results:**

CVS prevalence was 56.7% among students using printed textbooks and 58.7% among those using digital textbooks to study, with no significant differences between groups (aOR = 1.0; 95% CI, 0.6–1.7). Being female and having an uncorrected refractive error were associated with a higher likelihood of CVS.

**Conclusion:**

CVS prevalence was similar among students using digital and printed textbooks, and this pattern remained after adjustment for relevant covariates. However, appropriate optical compensation should be used when necessary to prevent an increase.

## 1 Introduction

Digital devices have become an essential component of adolescents’ daily lives, playing an increasing role in both personal, educational, and social activities. Among adolescents aged 10 to 15 years, 96.9% use computers, 96.5% access the Internet, and 67.9% own a smartphone (1). Smartphones are the most frequently used digital devices among adolescents due to their versatility and accessibility, integrating multiple functions such as virtual communication, access to online services, and use as entertainment and gaming platforms (2). Similarly, social media use is highly prevalent among adolescents aged 12 to 17 years, with entertainment, following trends, and meeting new people being some of its main purposes (3).

In addition to their widespread recreational use, digital devices have become increasingly integrated into educational environments. The incorporation of virtual learning environments in schools, such as the use of tablets to replace printed textbooks, is becoming increasingly common (4). These devices provide several advantages, such as a more attractive and interactive format for young people, large storage capacity, ease of transport, lower environmental impact, and greater economic savings (5). However, despite these benefits, increased screen exposure has raised concerns regarding its potential effects on ocular and visual health. Compared with printed texts, digital screens introduce additional visual demands, such as exposure to emitted light, glare, and reduced contrast, which may contribute to symptoms of asthenopia (6). In addition, prolonged exposure to digital devices is associated with alterations in the ocular surface, caused by a decrease in the frequency of spontaneous blinking and an increase in incomplete blink, thereby contributing to tear film disruption and ocular discomfort (7). These changes may manifest as ocular and visual symptoms, including itching, tearing, dryness, blurred vision, and headaches, which are collectively known as computer vision syndrome (CVS) or digital eye strain (8).

Most studies on CVS have focused on adults exposed to digital devices. Reported prevalence rates range from 42 to 97% among university students and from 19 to 95% among occupational populations (9–12). Several factors have been associated with an increased risk of CVS, including excessive digital device use, insufficient breaks, female sex, age, contact lens wear, uncorrected refractive errors, certain ocular pathologies, and accommodative or binocular dysfunctions. Additionally, environmental factors such as poor lighting, screen reflections, inappropriate viewing distances and postures, and unsuitable temperature and humidity conditions may further contribute to the development of CVS (13–19).

Despite the increasing exposure of younger populations to digital technologies, studies on CVS in adolescents are scarce. Most available evidence has been obtained from Asian populations and has reported a large variability in CVS prevalence, ranging from 12.1 to 94.8% (20). This heterogeneity is mainly due to the use of *ad hoc* questionnaires, not validated, or originally designed for other populations or languages (21–23).

Among the validated instruments available to assess CVS, the Computer Vision Syndrome Questionnaire (CVS-Q©) is one of the most widely used tools in adults (15, 18, 24–27). This questionnaire has been translated, culturally adapted, and validated in several languages (28–31). Recently, a Spanish version of the CVS-Q teen©, specifically adapted and validated for adolescents, has been developed, providing an appropriate instrument for assessing CVS symptoms in this population (32).

The impact of digital textbooks on CVS prevalence among high school students has not previously been assessed using validated tools. Accordingly, the aim of this study is to analyze the prevalence of CVS in adolescent students, comparing the use of printed versus digital textbooks, and considering sociodemographic, visual and digital device exposure factors.

## 2 Methods

### 2.1 Study design and target population

A longitudinal observational study was carried out with repeated assessments of the same participants and estimation of CVS prevalence at four time points: baseline (T0), time 1 (T1), time 2 (T2) and time 3 (T3). These time points were selected to capture periods of varying academic workload and avoid relying on a single period of particularly high or low academic demand, thereby providing a more representative estimate of digital device exposure throughout the academic year. Baseline was in February, T1 coincided with the period before Easter holidays, T2 just after Easter and T3 at the end of the academic course (33).

Target population included students of 4th compulsory secondary education (CSE) and 1st baccalaureate (BAC) (15–17 years) enrolled in two high schools (A and B) in the same town in the province of Alicante (Spain). In high school A, all students used only printed textbooks. In high school B, participation in a governmental program promoting the use of digital textbooks was optional; therefore, some students used tablets with digital textbooks, whereas others continued to use printed textbooks. Students selected their textbook format at the beginning of the academic year and maintained the same format throughout the entire academic year; therefore, their classification as digital or printed textbook users remained unchanged across the four assessments. Students with best-corrected monocular visual acuity worse than 0.0 logMAR after subjective refraction were excluded. Students who achieved 0.0 logMAR or better with subjective refraction but habitually wore no refractive correction or an outdated or inadequate correction were retained in the study and classified as having uncorrected refractive error. The presence of manifest ocular deviations and lack of fluency in Spanish were also considered exclusion criteria.

### 2.2 Data collection procedure

This study was approved by the Research Ethics Committee of the University of Alicante (UA-2020-01-13) and was conducted in accordance with the Declaration of Helsinki, Good Clinical Practice guidelines, and the applicable ethical principles for the protection of human subjects in biomedical research.

Parents or legal guardians of all students enrolled in the 4th year of compulsory secondary education (CSE) and the 1st year of baccalaureate (BAC) were informed about the study. Written informed consent was obtained from all participants and, where applicable, from their parents or legal guardians.

On the scheduled dates, two optometrists visited the participating high schools and coordinated the data collection procedures. Adolescents attended the designated assessment room individually according to a predetermined order. First, at baseline (T0), they completed two online questionnaires using laptops provided at the high schools (via Zoho Forms platform): an *ad hoc* questionnaire and digital device exposure, and the CVS-Q© teen questionnaire to assess CVS (32). The CVS-Q© teen is a 14-item self-administered questionnaire specifically developed and validated to assess ocular and visual symptoms associated with digital device use in adolescents. It evaluates both the frequency and intensity of each symptom. Frequency and intensity scores are subsequently recorded to determine the severity of each symptom, and the sum of these severity scores provides a total score. Adolescents with a total score of ≥ 6 are classified as having CVS.

Subsequently, all participants underwent a visual examination. Monocular and binocular visual acuity (VA) was assessed with each participant’s usual optical compensation at a viewing distance of 4 m using the Bailey-Lovie chart with Sloan letters. An automatic lensmeter (NIDEK LM-970, NIDEK Co., Ltd., Gamagori, Japan) was used to determine the optical prescription of participants wearing spectacles. Participants who wore contact lenses were asked in advance to bring their contact lens packaging or a photograph of the label to verify the lens type and prescription. For those who did not achieve a monocular VA ≤ 0.0 logMAR, objective refraction was performed by retinoscopy followed by subjective refraction using a trial frame and trial lenses. Clinical refraction data (S: sphere, C: cylinder, and α: axis) were converted into power vectors (M: spherical equivalent; J0 and J45: Jackson cross-cylinder components), and the “overall blurring strength” (B) was calculated as B = (M^2^ + J0^2^ + J45^2^)^1/2^. Participants were considered to have uncorrected refractive error if the difference in B between the subjective refraction and their usual optical compensation was greater than cero in at least one eye (34). Tracking and saccadic eye movements, pupillary reflexes were evaluated using the Hirschberg test, and cover test in near and distance vision were also assessed using prism bars to detect ocular deviations, always with the best optical compensation.

At T1, T2, and T3 students again completed the CVS-Q© teen and provided information on their exposure to digital devices.

### 2.3 Sample size calculation

The sample size was calculated assuming an expected prevalence of CVS of 60% among participants using printed textbooks and 75% among those using digital textbooks, with a two-sided significance level of 5% and 80% statistical power. A ratio of 1.5 printed textbook users for every digital textbook user was assumed. As each participant was assessed at four time points, the calculation accounted for the correlation between repeated measurements by assuming an exchangeable correlation structure and a within-participant correlation of 0.60 (35). Under these assumptions, a minimum of 215 participants was required. Allowing an additional 5% for possible absences, non-participation, or incomplete questionnaires, the target sample size was 227 participants. A total of 241 participants were included in the study.

### 2.4 Statistical analysis

Statistical analyses compared students using digital textbooks with those using printed textbooks, regardless of the high school attended. Logistic regression models were performed using generalized estimating equations (GEE) to account for the correlation between repeated observations from the same participant. Participant ID was specified as the clustering variable, with each student contributing up to four observations (T0–T3). A binomial distribution with a logit link and an exchangeable working correlation structure was specified, with robust standard errors.

Data were available for 231, 229, 230, and 230 participants at T0, T1, T2, and T3, respectively. Thus, only four of the 924 potential repeated observations (0.4%) were missing. All available observations were included in the GEE analyses. The prevalence of CVS presented in the tables was calculated as the mean of the prevalence estimates obtained at the four time points.

Analyses were first stratified by textbook type (printed or digital). Within each group, crude and adjusted associations between CVS and sex, age, educational institution, uncorrected refractive error, daily use of digital devices to study, weekly use of digital devices to study, and daily use of digital devices to leisure were estimated. These covariates were selected *a priori* based on their clinical and epidemiological relevance identified in previous literature. Multivariable models also included time point.

Finally, the association between textbook type and CVS was assessed in the overall sample using a multivariable GEE model adjusted for the same covariates and time point. Measures of association were expressed as crude odds ratios (cOR) and adjusted odds ratios (aOR) with their corresponding 95% confidence intervals (95% CI). Statistical analyses were performed using R 1.1.463 and Rstudio 3.6.0 (36).

## 3 Results

A total of 241 eligible students agreed to participate in the study. Of these, 10 were excluded (9 did not achieve the minimum VA criterion, and 1 had language difficulties). Finally, the sample consisted of 231 adolescents, aged 15.67 ± 0.66 (mean ± standard deviation). Of the participants, 51.9% were women. In high school A, all students (100%) used printed textbooks, whereas in high school B, 34.3% used printed textbooks and 65.7% used digital textbooks. Overall, 61% of the total sample used printed textbooks and 39% used digital textbooks. No statistically significant differences were observed between both groups, except in the use of digital devices for studying, being higher in the group using digital textbooks compared to the group using printed textbooks, both in hours per day and days per week (*p* < 0.001) (Table 1).

**Table 1:** Sociodemographic, visual, and digital device exposure characteristics of study participants (*n* = 231) according to the use of printed or digital textbooks.

Variable	Printed		Digital		*p* value
	*n*	%	*n*	%	
Sex					
Male	61	55.0	50	45.0	0.08
Female	80	66.7	40	33.3	
Academic year					
4th CSE	88	57.9	64	42.1	0.20
1st BAC	53	67.1	26	32.9	
Educational institution					
A	94	100.0	0	0.0	**<0.001**
B^†^	47	34.3	90	65.7	
Uncorrected refractive error					
No	110	62.5	66	37.5	0.43
Yes	31	56.4	24	43.6	
Daily use of digital devices to study (hours/day)					
≤4	124	67.0	61	33.3	**<0.001**
>4	17	37.0	29	63.0	
Weekly use of digital devices to study (days/week)					
≤4	77	87.5	11	12.5	**<0.001**
>4	64	44.8	79	55.2	
Daily use of digital devices to leisure (hours/day)					
≤4.5	72	56.7	55	43.3	0.14
>4.5	69	66.3	35	33.7	
Total					
	141	61.0	90	39.0	

CSE: compulsory secondary education (15–16 years); BAC: baccalaureate (16–17 years). Bold: *p* value < 0.05.

†Educational institution B was part of the government’s program to promote the use of digital textbooks.

No statistically significant differences in CVS prevalence were observed between students using printed and digital textbooks (56.7% vs. 58.7%, respectively; aOR = 1.0; 95% CI, 0.6–1.7). After adjustment for all variables, female sex was associated with a higher odds of CVS in both the printed textbook group (aOR = 2.8; 95% CI, 1.5–5.1) and the digital textbook group (aOR = 5.5; 95% CI, 2.5–11.9). Uncorrected refractive error was also associated with CVS among students using digital textbooks (aOR = 4.0; 95% CI, 1.6–9.5), whereas no statistically significant association was observed among students using printed textbooks. No statistically significant association was observed between time spent using digital devices for study or leisure and CVS in either group (Table 2).

**Table 2:** Prevalence of computer vision syndrome and its association with characteristics according to the use of printed or digital textbooks.

Variable	P (%)	Printed				P (%)	Digital			
		cOR	95% CI	aOR	95% CI		cOR	95% CI	aOR	95% CI
Sex										
Male	44.9	Ref		Ref		40.5	Ref		Ref	
Female	69.4	2.8	[1.6–5.1]	2.8	[1.5–5.1]	76.9	4.9	[2.3–10.4]	5.5	[2.5–11.9]
Uncorrected refractive error										
No	55.6	Ref		Ref		50.0	Ref		Ref	
Yes	70.0	1.8	[0.9–3.7]	1.6	[0.7–3.4]	75.0	3.0	[1.2–7.2]	4.0	[1.6–9.5]
Daily use of digital devices to study (hours/day)										
≤4	58.1	Ref		Ref		51.2	Ref		Ref	
>4	63.2	1.1	[0.8–1.4]	1.0	[0.7–1.4]	68.1	1.0	[0.7–1.5]	0.9	[0.5–1.4]
Weekly use of digital devices to study (days/week)										
≤4	55.1	Ref		Ref		61.3	Ref		Ref	
>4	63.8	1.2	[0.9–1.5]	1.1	[0.8–1.4]	53.1	1.3	[0.9–1.8]	1.0	[0.6–1.5]
Daily use of digital devices to leisure (hours/day)										
≤4.5	54.4	Ref		Ref		51.8	Ref		Ref	
>4.5	63.2	1.1	[0.8–1.5]	1.1	[0.8–1.5]	61.0	1.2	[0.8–1.7]	1.4	[0.8–2.2]

P: prevalence; cOR: crude odds ratio; aOR: adjusted odds ratio for all variables; 95% CI: 95% confidence interval.

## 4 Discussion

The present study found no higher prevalence of CVS among adolescent students using digital textbooks on tablets compared with those using printed textbooks. Students in the digital textbook group spent, on average, more hours per day using digital devices for studying. However, leisure-related digital device use was slightly higher among students in the printed textbook group, although this difference did not reach statistical significance. Being female is associated with CVS regardless of the type of textbook used, whereas uncorrected refractive error was associated with CVS only among students using digital textbooks. Whereas the amount of time spent using digital devices for studying or leisure activities is not associated with CVS.

On the one hand, these findings suggest that educational models incorporating digital devices as the primary learning tool may not, by themselves, be sufficient to increase the risk of CVS compared with traditional learning environments using printed textbooks. In contrast, other individual and environmental factors may play a more important role in the development of symptoms. This observation is consistent with current evidence indicating that CVS is a multifactorial condition influenced not only by screen exposure time but also by sociodemographic, ergonomic, visual, and environmental determinants (37).

A higher prevalence of CVS has been reported among university students, with 76.6% of participants affected, as assessed using the same methodology (24). This difference may be explained by several factors. First, our study applied more restrictive exclusion criteria regarding monocular and binocular visual function, which may have reduced the inclusion of students with pre-existing visual conditions that could contribute to CVS symptoms. Second, adolescents may have lower cumulative exposure to digital devices than university students. In the aforementioned study, university students reported a mean daily digital device of 9.0 ± 3.7 h, compared with 7.41 ± 3.22 among adolescent students in the present study. Recent systematic review has shown that university students represent one of the populations with the highest reported prevalence of CVS, likely due to intensive digital device use and sustained near visual demands (20).

The prevalence of CVS observed in our study was slightly higher than that reported by Abuallut et al. among adolescents aged 16–18 years (54.1%) (38). Nevertheless, this difference was minimal and may be explained by the use of the original CVS-Q©, which was validated for adults, rather than the CVS-Q© teen. In addition, the authors did not report on adolescents’ daily digital device exposure, limiting the ability to make well-founded comparisons with our findings.

Another novel finding is the high risk of CVS in females, even from adolescence, which is observed regardless of the type of textbook used. This result agrees with previous studies identifying female sex as one of the most consistent predictors of CVS (18, 20, 26). As in the adult population (13, 39, 40), this association could be due to a greater tendency among women to report symptoms and to the hormonal fluctuations typical of this stage, which could affect tear production and stability (41).

On the other hand, uncorrected refractive errors have previously been identified as a factor associated with CVS in adult populations (42), possibly because the additional visual effort required to maintain clear vision may contribute to ocular and visual symptoms (43). In our stratified analyses, uncorrected refractive error was associated with CVS among students using digital textbooks, whereas no statistically significant association was observed among those using printed textbooks. However, these findings should be interpreted cautiously, as the presence of a significant association in one group and not in the other does not necessarily indicate that the association differs between groups in the absence of a formal interaction analysis.

The absence of an association between digital device exposure time and CVS symptoms suggests that the duration of screen exposure alone may not adequately reflect the visual demands associated with digital device use. Ergonomic and environmental factors, such as viewing distance, posture, lighting conditions, screen reflections, and the frequency of breaks, may also influence the development of CVS (13, 16, 18, 37). However, these factors could not be directly assessed in either the school or home environments, representing an inherent limitation of this study. Future studies incorporating objective measurements of these factors would help clarify the relative contribution of educational screen exposure and modifiable environmental determinants to CVS in adolescents. In addition, they may help explain the stronger association observed between uncorrected refractive errors and CVS among students using digital textbooks, as differences in ergonomic conditions between digital and printed textbook use could partly account for this finding. Such evidence would be valuable for developing evidence-based recommendations aimed at promoting healthy digital learning environments in schools.

Our study had several additional limitations. Participants were recruited from only two high schools located in the same town, which may limit the generalizability of the findings to adolescents from other geographical, socioeconomic, or educational settings. In addition, despite adjustment for the variables considered relevant in the analyses, residual confounding due to unmeasured or insufficiently measured factors, such as the ergonomic and environmental factors discussed in the previous paragraph, cannot be ruled out. Moreover, digital device exposure was self-reported, which may have introduced some degree of recall or reporting bias; however, self-reported exposure is the approach most commonly used to assess digital device use in studies of this type. Finally, given the observational nature of the study, the associations identified cannot be interpreted as causal relationships.

This study has several strengths that should be highlighted. First, CVS was assessed using the CVS-Q© teen, a validated patient-reported outcome measure (PROM) specifically designed for adolescents. The use of a standardized, age-specific instrument enhances the precision of symptom assessment and supports meaningful comparisons across adolescent populations in future research. In addition, because the questionnaire is self-reported, it captures the adolescents’ own perception of their visual symptomatology, avoiding the potential bias associated with proxy reporting by parents or legal guardians. Finally, by performing repeated assessments throughout the academic year, we sought to minimize the influence of temporal variations in digital device use, which may fluctuate according to academic demands, as well as other factors that could affect CVS symptoms.

Overall, these findings suggest that CVS prevalence was similar among students using digital and printed textbooks, and this pattern remained after adjustment for relevant covariates. Female sex was consistently identified as an important factor associated with a higher likelihood of CVS in both educational contexts. Furthermore, the presence of uncorrected refractive errors may represent a relevant contributing factor, particularly among students using digital textbooks, highlighting the importance of adequate visual assessment and correction in adolescents exposed to digital learning environments.

## Data Availability

The raw data supporting the conclusions of this article will be made available by the authors, without undue reservation.
